# Application of a Static Fluorescence-based Cytometer (the CellScan) in Basic Cytometric Studies, Clinical Pharmacology, Oncology and Clinical Immunology

**DOI:** 10.1080/17402520500160895

**Published:** 2005-09

**Authors:** Michal Harel, Boris Gilburd, Yael S. Schiffenbauer, Yehuda Shoenfeld

**Affiliations:** 1 Center for Autoimmune Diseases Department of Medicine B Chaim Sheba Medical Center (Affiliated to Sakler Faculty of Medicine, Tel-Aviv University) Tel-Hashomer Israel; 2 Medis-El Ltd Yehud 52621 Israel; 3 Incumbent of the Laura Schwarz-Kipp Chair for Research of Autoimmune Diseases Tel-Aviv University Tel-Aviv Israel

## Abstract

The CellScan apparatus is a laser scanning cytometer enabling repetitive fluorescence intensity (FI) and polarization (FP) measurements in living cells, as a means of monitoring lymphocyte activation. The CellScan may serve as a tool for diagnosis of rheumatoid arthritis (RA) and systemic lupus erythematosus (SLE) as well as other autoimmune diseases by monitoring FP changes in peripheral blood lymphocytes (PBLs) following exposure to autoantigenic stimuli. Changes in FI and FP in atherosclerotic patients' PBLs following exposure to various stimuli have established the role of the immune system in atherosclerotic disease. The CellScan has been evaluated as a diagnostic tool for drug-allergy, based on FP reduction in PBLs following incubation with allergenic drugs. FI and FP changes in cancer cells have been found to be well correlated with the cytotoxic effect of anti-neoplastic drugs. In conclusion, the CellScan has a variety of applications in cell biology, immunology, cancer research and clinical pharmacology.


*Clinical & Developmental Immunology*, September 2005; 12(3): 187–195

### Application of a static fluorescence-based cytometer (the CellScan) in basic cytometric studies, clinical pharmacology, oncology and clinical immunology

MICHAL HAREL[1], BORIS GILBURD[1], YAEL S. SCHIFFENBAUER[2,†], & YEHUDA SHOENFELD[1,3,‡]

[1]*Center for Autoimmune Diseases, Department of Medicine B, Chaim Sheba Medical Center (Affiliated to Sakler Faculty of Medicine, Tel-Aviv University), Tel-Hashomer 52621, Israel,* [2]*Medis-El Ltd., Yehud 56101, Israel, and* [3]*Incumbent of the Laura Schwarz-Kipp Chair for Research of Autoimmune Diseases, Tel-Aviv University, Tel-Aviv, Israel*

**Abstract**
The CellScan apparatus is a laser scanning cytometer enabling repetitive fluorescence intensity (FI) and polarization (FP) measurements in living cells, as a means of monitoring lymphocyte activation. The CellScan may serve as a tool for diagnosis of rheumatoid arthritis (RA) and systemic lupus erythematosus (SLE) as well as other autoimmune diseases by monitoring FP changes in peripheral blood lymphocytes (PBLs) following exposure to autoantigenic stimuli. Changes in FI and FP in atherosclerotic patients' PBLs following exposure to various stimuli have established the role of the immune system in atherosclerotic disease. The CellScan has been evaluated as a diagnostic tool for drug-allergy, based on FP reduction in PBLs following incubation with allergenic drugs. FI and FP changes in cancer cells have been found to be well correlated with the cytotoxic effect of anti-neoplastic drugs. In conclusion, the CellScan has a variety of applications in cell biology, immunology, cancer research and clinical pharmacology.

**Keywords:** *Autoimmunity, CellScan, fluorescence polarization, rheumatoid arthritis, systemic lupus erythematosus, atherosclerosis*

### Introduction

The CellScan apparatus is a laser scanning cytometer incorporating a unique cell carrier that allows repeated high-precision fluorescence intensity (FI) and polarization (FP) measurements to be made on intact living cells under physiological conditions (Zurgil et al. 1999a). The fixed location of the cells in the carrier permits kinetic measurements, re-measurement after reaction with a stimulant or drug, or monitoring the same population under a different machine set-up (Kaplan et al. 1997). Reviewed here are the different implementations of the CellScan system in immunology, cell biology, cancer research and clinical pharmacology.

The processes linking early and late intracellular events in the course of cell activation and apoptosis involve conformational changes of cytosolic enzymes and/or their regulatory proteins, as well as their intracellular matrix reorganization. The monitoring of these early and structural changes can be preformed by measuring the FP of intracellular fluorescence probes (Deutsch et al. 2000). Among these probes is fluorescein diactetate (FDA), a non-fluorescent molecule that undergoes fluorochromasia in viable cells. This includes penetration of FDA, its enzymatical hydrolysis, which converts it to fluorescein, and the accumulation of the latter (Zurgil et al. 1996).

Correspondence: Y. Shoenfeld, Department of Medicine 'B' and Center for Autoimmune Diseases, Sheba Medical Center, Tel-Hashomer 52621, Israel. Tel: 972 3 5302652; Mob: 972 52 666120. Fax: 972 3 5352855. E-mail: shoenfel@sheba.health.gov.il

[†] Yael S. Schiffenbauer is an employee of Medis-El Ltd.
[‡] Yehuda Shoenfeld is a consultant to Medis-El Ltd.

ISSN 1740-2522 print/ISSN 1740-2530 online © 2005 Taylor & Francis
DOI: 10.1080/17402520500160895

Upon excitation with a polarized light, FP reflects the level of freedom of the intracellular dye, due to its interaction with various cellular components in different intracellular oraganelles (Fixler et al. 1997). The more the molecule rotational movement is restricted, the higher the FP value, and vice versa. If molecules are free to change their orientation randomly, then due to the time lapse between excitation and emission, the polarization of the fluorescence relative to the excitation light changes randomly, and decreases (Zurgil et al. 2002). Changes in FP have been detected concurrently with physical alterations of the cells: Changes in osmolarity, cell stimulation, apoptosis and progression through cell-cycle phases (Deutsch et al. 2000).

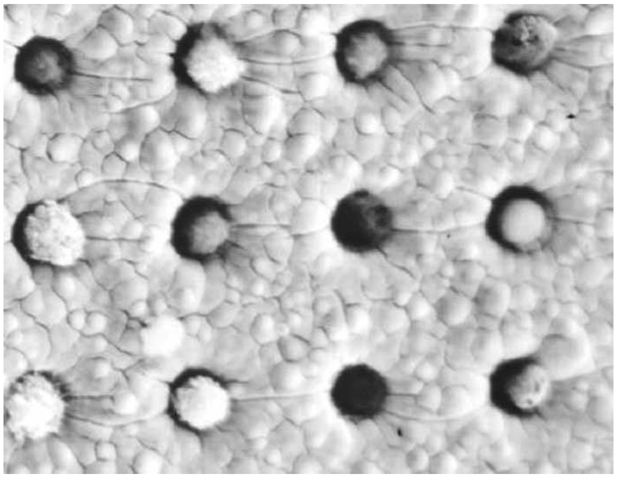

Figure 1. An electron micrograph of lymphocytes trapped on the Cellcarrier.

### The CellScan apparatus

The CellScan apparatus was developed with the objective of enabling repetitive FP measurements on individual cells. The apparatus has three principal components:

(a) A cell carrier, consisting of a grid of precisely dimensioned and contoured microscopic holes on a flat surface, which captures cells of a desired size and holds them in place for repeated measurements. The carrier, used for lymphocytes, has holes 5–8 μm in diameter, spaced 20 μm apart; a 2-mm squared grid can hold 10,000 cells, each loaded in a separate trap (Figure 1).
(b) A motor-driven, microscopic stage that holds and moves the cell-carrier.
(c) A micro-photometer comprised of a blue laser light source, microscopic optics for illuminating the sample and collecting fluorescent light, and four photomultiplier tubes and associated optical and electronic equipment for measuring FP at two wavelengths.

Three parameters are considered in measuring fluorescence emission from cells: FI, FP, and measurement time ($t$). Classification of subgroups of cells may be based on any of these parameters. Acquired data is displayed on-line, both graphically and numerically (Figure 2). The software determines all test limits, before, during and after the scan. All on-line statistics are displayed during the test, as well as numerous numerical and graphic screens, e.g. historgrams and scatter diagrams (Deutsch et al. 2000).

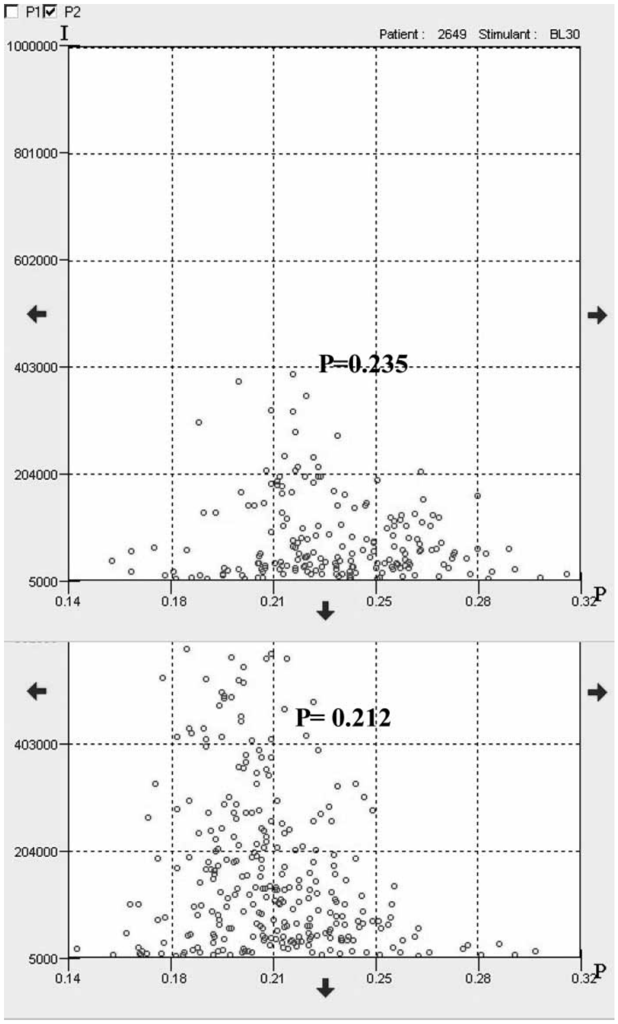

Figure 2. Scatter plots of FI (vertical axis) vs. FP (horizontal axis) of control (A) and PHA-treated lymphocytes (B). The drop in average FP (marked P in the center of the figure) of PHA-treated lymphocytes is observable.

### Monitoring lymphocyte biology

FP of Jurkat T-lymphocytes has been demonstrated to be cell-cycle dependent. Phase selection was achieved either by differential cell densities or phase-arresting drugs such as hydroxyurea and noconazole. As the cell density and percentage of cells in the resting phases $G_0/G_1$ stages had increased, a concomitant and significant increase in FP was observed. Both hydroxyurea mediated S phase arrest and noconazole mediated $G_2/M$ arrest induced a reduction in FP. Thus, FP is cell-cycle dependent with significantly lower values for cycling than for non-cycling populations (Zurgil et al. 1996).

It has been established that thiols are of prime importance to the functional performance of lymphocytes. The CellScan apparatus in conjunction with the probe chloromethyl fluorescein diacetate (CMFDA), have been used to monitor thiol levels in individual cells responding to a variety of experimental conditions. The analysis of FP of intracellular fluorophore fluorescence signals has been used as a tool for discriminating between unconjugated 5-chloromethyl fluorescein (CMF), the hydrolyzed product of CMFDA, and a thiol bound adduct. Also, the covalent conjunction of intracellular fluorescent molecules to sulphydryl residues was revealed by the use of the FP ratio. The proportion of SH-F adduct and the unconjugated CMF in the cell has been found to be modulated by parameters such as CMFDA concentration, staining duration as well as the elapsed time following the cessation of the staining process. An increase in intracellular thiol levels has been observed during cell proliferation, attributed to the oxidative activity following stimulation (Zurgil et al. 1999b).

### Monitoring lymphocyte activation

T-cell stimulation is an induced physiological process of major importance in a wide range of immunological responses. It involves a cascade of biochemical and biophysical cellular events initiated by antigen recognition followed by transmission of signals to the nucleus, ultimately leading to proliferation (Kaplan et al. 1997).

Studies have shown that lymphocyte activation is accompanied by alterations in the structuredness of the cytoplasmic matrix (SCM) causing changes in FP. Traditionally, T-cell activation is assayed by determining lymphoblast formation in culture, by measuring the incorporation of radioactive DNA precursors. Another means has been the monitoring of cytoplasmic $Ca^{++}$ increase following stimulation. FP monitoring has advantages over both methods; Unlike DNA precursor incorporation that measures cell proliferation commencing 24 h after stimulation, changes in FP appear at a very early stage of stimulation (50 min within onset of stimulation). Also, while elevated $[Ca^{++}]i$ is transient and difficult to follow, FP changes are stable for hours.

Using the CellScan, the alterations in SCM following stimulation were measured by monitoring FP of FDA-labeled cells. Exposure of T-lymphocytes to polyclonal proliferation inducers, such as the lectin phytohaemagglutinin (PHA), has been shown to cause a concentration-dependent decrease in FP (Kaplan et al. 1997).

Using this stimulation method, it has been found that cells within a mixed population can be distinguished both by their individual FI and by FP kinetics. Although great variance was noted, a characteristic phenomenon was observed in which the cell-staining rate may be divided into three major subgroups: low (L), medium (M) and high (H). A decrease in FP induced by PHA was most prominent in the low subgroup, while the high subgroup did not show such changes, and even yielded an increase in FP. All groups showed a decrease in FP values over time, but while the L subgroup quantitatively conserved fluorescence depolarization (FDP), in the other subgroups (H and M) the FDP decreased or even changed sign. The distinction between these three groups is therefore important in the prediction of FP changes following stimuli (Sunray et al. 1999).

A similar decrease in FP was observed in lymphocytes exposed to concanavalin (ConA) and pokeweed mitogen (PWM), T and B-lymphocytes stimulants, respectively, although to a lesser extent than following PHA incubation, as represented by the length of the incubation period and the minimal concentration needed for stimulation.

A decrease in FP was also shown in $CD3^{+}$ lymphocytes after incubation with anti-CD3 antibody (Eisenthal et al. 1996), and in lymphocytes exposed to phorbol 12-myristate 13-actetate (PMA), a phorbol ester which activates protein kinase C (PKC). This last finding suggests the decrease in FP upon stimulation reflects intracellular changes in the pathway of PKC induction (Kaplan et al. 1997).

Using the CellScan, the biological significance of changes in FP in PBLs exposed to PHA and anti-CD3 has been further investigated. It has been established that the extent of polymerization of the cytoskeleton microtubules and microfilaments is affected during cell activation. Therefore, cytochalasin B, a microfilament structure modulator, as well as vinblastine and cholchicine, which both affect the microtubules, were employed in order to analyze their effect on FP of stimulated cells. Colchicine, vinblastine and cytochalasin B added to PHA or anti-CD3 stimulated PBLs have been shown to reverse the effect of these stimulants on FP, as well as inhibit DNA synthesis. The similarity by which these cytoskeleton modulators affected both skeleton polymerization and cell proliferation points toward the possible role of the cytoskeleton in signal transduction, as well as further establish their role in cell activation (Eisenthal et al. 1996).

A similar research using human lung fibroblasts has shown that exposure of the fibroblasts to interleukin 1$\alpha$, interleukin 1$\beta$ and tumor necrosis factor $\alpha$, which are known to be fibroblast-cell activators as well as modulators of immune responses, induced a decrease in FP. This effect was reversed in the presence of vinblastine and cytochalasin (Marder et al. 1996), suggesting that the role of microtubules and microfilaments in cell activation is not lymphocyte-specific, but rather a common denominator of cell activation.

It has been demonstrated that infection of human PBLs with influenza A virus, a known modulator of the immune response, reduces FP. It has been suggested that viral infection induces cellular activation, which affects the cytoskeleton. In addition to the effect observed in resting PBLs, influenza A virus has synergistically decreased FP following exposure to PHA, further establishing the role of the virus as a cytoskeletal modulator. The decrease in FP induced by the virus has also lead to an increase in cell proliferation following incubation with PHA, compared to non infected cells, in a dose-dependent manner. These results confirm the effect of the virus on cell activation via modulation of the cytoskeleton.

The mechanism by which the influenza virus alters the cytoskeleton is yet unknown. One possibility is that viral proteins, induced during infection, affect monomeric G actin, thus affecting cytoskeletal polymerization. This hypothesis is supported by the correlation between the kinetics of FP changes and the time range of H1 viral protein induction (Eisenthal et al. 1997).

Recombinant soluble peptide-single-chain major histocompatibility complex (MHC) tetramers are valuable reagents for characterizing immune responses involving T cells. The CellScan has been used to monitor the binding of melanoma specific-MHC tetramers to T-cells through monitoring of FI, complemented by the assessment of changes in FP, to detect the response of T lymphocytes. The findings were well correlated with lymphocyte binding and stimulation as confirmed by FACS analysis and INF-$\gamma$ release test, respectively. The ability to monitor both binding and lymphocyte stimulation makes the CellScan system a useful tool in detection of responsive T-cells in various immunological settings (Cohen et al. 2003).

### Monitoring of effector and target cell interaction

FP measurements have been used to demonstrate prelytic intracellular changes induced in target and effector cells following conjugation at room temperature. It has been shown that conjugation of K562 cell lines with natural killer (NK) cells or with lymphocyte activated killer (LAK) cells yielded a decrease in FP. These results were in agreement with the results obtained using the Chromium release assay used to monitor cell-killing activity of NK and LAK cells. The findings were also consistent with the expression of ICAM-1 on K562 lymphocytes as indicated by phenotyping analysis. Since ICAM was shown to serve as an adhesion molecule regulating the binding of NK cells to their target, these results further strengthen the role of ICAM in the FP changes induced during NK and target cell interaction (Fixler et al. 1998).

FP has been used to determine the role of cytoskeletal changes in target and effector cells infected by infulenza A virus. It has been shown that infection of both K562 target cells and NK effector cells causes an increase in cell lysis. This increase has been well correlated with an increase of ICAM-1 expression. FP measurements have shown changes in the cytoskeleton following infection of K562 cells by influenza A virus, indicating the involvement of such changes in the regulation of NK lysis. One possibility is that the cytoskeletal changes may result in reorganization of ICAM forming a uropodson, a bud-like cellular projection, which may render NK target cells more susceptible to killing through this ligand (Eisenthal et al. 1997).

Separate specific staining and FP monitoring of effector and target cells shows early reciprocal and specific stimulation responses of K562 cells in conjugation with NK/LAK cells. These measurements also indicate the time-dependent nature of stimulation. Two phases were described. In the first phase, a marked sharp depolarization followed by a slow repolarization was observed in both the target and effector cells. In the second phase, the FP of the effector cell remains in its control value while that of the target cell continuously decreases. While the first phase is attributed to conjugation-related stimulation of both cells, it is suggested that the second phase indicates the target cell commitment to a lethal trajectory. The kinetics of FP measurements indicate that the stimulation of NK cells due to conjugation precedes the reciprocal stimulation of K562 cells by a few minutes, later becoming lethal.

Also, these results are consistent with a dynamic structural orientation of intracellular components during cell activation, indicating an initial reciprocal orientation followed by a vertical one (Fixler et al. 1997).

### Monitoring apoptotic events

Apoptosis, or regulated cell death, is a physiological elimination process of damaged or unwanted cells, which ensures equilibrium between cell proliferation and death. While a low level of apoptosis has been linked to malignancies, resistance to anti-cancer drugs, and autoimmune diseases, a high level of apoptotic activity can result in immune deficiency and degenerative conditions. Hence, the measurement of cellular apoptotic activity has become a matter of great importance in the diagnosis and monitoring of these conditions (Zurgil et al. 2000).

Evidence has been provided that the hyper polarization (HP) of intracellular fluorescein can be utilized as an early kinetic marker for apoptosis detection. The increase in the FP parameter was found to be ubiquitous, since it was demonstrated in several well-known models of cells undergoing apoptosis by various agents (i.e. serum-deprived and dexamethasone treated Jurkat T cells, cultured, dexamethasone and anti-Fas treated mouse thymocytes).

The intracellular HP coincides with morphological changes observed by a light diffraction cytometer. A direct correlation was found between the FP changes and the decrease in cell diameter. In addition, a decrease in FI was measured concurrently with FP changes of apoptotic cells. HP in FDA labeled cells has been shown to appear earlier than the phosphatidylserine exposure and to be a more sensitive measure for early apoptosis in both mouse thymocytes and Jurkat T-cells (Zurgil et al. 2000).

It has been found that the cytoplasmic condensation, reflected by the increase in FP, preceded other functional apoptotic parameters. The change in intracellular FP can be modulated by osmolarity changes in the medium, suggesting that cell dehydration and shrinkage may be involved in the phenomena of intracellular fluorescein HP in apoptosis. Caspase inhibitors exhibited effect neither on HP nor on volume decrease during the early stages of apoptosis, suggesting that cell shrinkage precedes caspase activation.

Measurements of changes in FI of the fluophore acridine orange (AO) at two wavelengths indicated early alterations in lysosomal stability in Jurakt cells subjected to mild oxidative stress. These findings support the information regarding the involvement of lysosomal rupture in oxidative stress induced apoptosis (Zurgil et al. 2002).

FP measurements have been used to monitor apoptotic processes induced by oxidized low-density lipoprotein (oxLDL) in T-lymphocytes and monocytes cell-lines. OxLDL, which plays a key role in atherogenesis, has been found to be cytotoxic to both cell types, in a time- and dose-dependent manner. These findings have been demonstrated by early cytoplasmic condensation resulting from cell shrinkage, as measured by an increase in FP of fluorescein-labeled cells. The radical scavenger, superoxide dismutase (SOD), reduced the effect of ox-LDL in a time- and dose-dependent manner. The hyperpolarization of fluorescein-labeled cells has preceded the appearance of phosphatidylserine on the plasma membrane. FP measurements have detected different cell death kinetics, as well as varying sensitivity to the inhibitory effect of SOD in monocytes and lymphocytes, suggesting that reactive oxygen species generation are involved in ox-LDL-induced apoptosis. It has also been demonstrated that monocytes are more susceptible to cell death triggered by oxidative stress. Thus, FP measurement may serve as a research tool in the pathogenesis of atherosclerosis, specifically through apoptotic cell death (Zurgil et al. 2004).

### Analysis of enzyme kinetics

The CellScan ability to monitor time kinetic events through repetitive measurement has been employed in the study of intracellular enzymatic kinetics. The apparatus was used to monitor the esterase hydrolysis of two flourogenic substrates, FDA and CMFDA, which differ in their kinetics. The FI curve of FDA-labeled cells was used to calculate the Michaelis-Menten constant $K_m$, which was similar in different cell populations. Such similarity was not found in FI curves of CMFDA-labeled cell populations, although their FP kinetic behavior was very similar. FI depends on many variables, irrelevant of the enzymatic process. Therefore, measuring FP may be used as an additional tool to monitor enzymatic processes, and thus contribute to their understanding (Deutsch et al. 2000).

Monitoring the FI kinetics of the FDA enzymatic hydrolysis was also applied to trace differences in distributions of $K_m$ and $V_{max}$ over a population of individual lymphocytes that were incubated with or without the mitogen PHA. It has been found that average $K_m$ and $V_{max}$ values profoundly increase following PHA-stimulation. These changes in $K_m$ and $V_{max}$ have appeared to be entirely due to an increase in the dissociation rate $k_2$. This supposition is well supported by both theory and experimental findings concerning changes in intracellular pH, viscosity and cell diameter upon stimulation. These findings further establish the CellScan as a tool for assessing enzymatic reaction kinetics (Sunray et al. 2002).

Such cellular enzymatic activity measurements have been applied to the monitoring of breast cancer. Steady state measurements of FI of FDA labeled cells have been used to demonstrate the $K_m$ and $V_{max}$ of peripheral blood cell esteraze-activity. A marked difference in such enzymatic characteristics has been shown between breast cancer patients and healthy individuals following exposure to tumor tissue. In addition, it has been found that higher $K_m$ values are associated with a better prognostic status of breast cancer (lymph node-negative tumors, hormone receptor preservation, and the absence of Her-2/*neu* protein over-expression). Thus, the $K_m$ and $V_{max}$ of peripheral blood monocyte reactions may serve as additional criteria for monitoring and prognosis of breast cancer (Afrimzon et al. 2004).

### Early cancer detection

Early detection of cancer is of crucial importance to the patient's life duration and quality, prognosis and treatment options. FP measurement, or the SCM test, has been first suggested by Cercek and Cercek (1997)

as a screening tool for neoplastic diseases. This approach is based on the appearance of memory lymphocytes in the circulation, which recognize antigens expressed by tumor cells. Hence, exposure of these memory cells to cancer-related antigens may then induce stimulation detected by the changes in FP (Merimsky et al. 1996).

It has been reported additionally that lymphocytes respond to stimulation with a tumor-derived antigen in an organ site-dependent fashion. PBLs from patients with breast cancer show decrease in polarization following exposure to substances from breast cancer tissue or cancer cells while not responding to substances derived from colon or lung cancer. This, in principle, should permit determination of the primary site of tumors diagnosed by the SCM test. The SCM test was controversial at first due to technical complexities that made its clinical application difficult (Deutsch et al. 1996). The CellScan apparatus simplifies and increases the precision of the FP measurements, and also allows the analysis to be made on lymphocytes isolated by a relatively simple procedure (Rahmani et al. 1996), thus making the SCM test a more clinically applicable option.

The SCM test and the CellScan have been shown to distinguish between different neoplastic diseases-patients and healthy controls. Among the neoplasms studied were colorectal cancer (Merimsky et al. 1996), breast cancer (Rahmani et al. 1996, Ron et al. 1995, 1996, Klein et al. 2002a, 2002b), prostate cancer (Klein et al. 1999), malignant melanoma (Merimsky et al. 1997), lung and ovarian cancer (Deutsch et al. 1996). In these studies, diagnostic algorithms have been created, based on PBLs responses to different antigenic stimuli. These stimulants include PHA, cancer basic protein (CaBP), encephalogenic factor (EF), and tumor-associated antigens (TAA) for the detection of colorectal and breast cancer (Merimsky et al. 1996, Deutsch et al. 1996, Rahmani et al. 1996). No significant stage-related differences were found in the polarization values following stimulation by EF or PHA in breast cancer patients (Ron et al. 1996). Also used were CaBr (Ron et al. 1995) and MUC-1/SEC (Klein et al. 2002a, 2002b), both tumor-associated antigens specific for breast cancer, PSA-ACT, a prostate specific antigen (Klein et al. 1999), MEL, a melanoma antigen, as well as different tumor antigen extracts (TAE) (Deutsch et al. 1996, Rahmani et al. 1996).

Using the diagnostic algorithms, sensitivity and specificity values achieved have ranged between 60–97% and 80–98% respectively.

### Monitoring autoimmune phenomena

T-lymphocytes with the capacity to respond to organ-specific autoantigens, including anti-self T-cells with pathogenic potential, are present in the normal immune repertoire. These anti-self T-cells can be activated by exposure both to the corresponding auto-antigen and by co-stimulatory signals. It has been found that such T-cells mediate autoimmune response in some of the human autoimmune disorders (Zurgil et al. 1997).

*Rheumatoid arthritis* (*RA*) is a multi system disease associated with impaired cellular immune function. Many studies have demonstrated the role of T-lymphocytes in the pathogenesis of RA. Joint-derived T-cells of RA patients were shown to react with self-immunoglobulin heavy chains or immunoglobulin-binding proteins. Using the CellScan, it has been shown that incubation of PBLs derived from RA patients with affinity purified IgG resulted in a reduction of FP. Kinetic analysis of the FP decay of RA-lymphocytes has shown an exponential pattern typical of resting lymphocytes prior to incubation with IgG, and a linear pattern typical of PHA stimulated lymphocytes following the incubation.

In addition, it has been shown that most of the rheumatoid factor (RF)-negative patients could be detected by testing with the CellScan apparatus. Thus, the CellScan may allow better diagnosis and disease monitoring of RA patients who are RF-negative, a group which accounts for 25% of adult RA patients and 90–95% of juvenile RA patients (Zurgil et al. 1999c).

*Systemic lupus erythematosus* (*SLE*) is a systemic autoimmune disease characterized by the production of autoantibodies (autoAb) directed against cell-surface, nuclear and cytoplasmic proteins. It has been proven that nucleosomes are major autoantigens for the pathogenic activation of T and B-lymphocytes in SLE. The analysis of sensitivity and specificity of autoAb directed against native nucleosomes in patients with autoimmune disease has demonstrated high prevalence, clinical specificity, and diagnosis confidence in patients with SLE. Moreover, these antibodies are linked to disease activity and may contribute to the development of lupus nephritis. It has been found that FP levels in FDA-stained cells of SLE patients detected by the CellScan decrease following stimulation by a nucleosomal autoantigen. This fluorescence depolarization was autoantigen-specific and dose-dependent, as well as highly specific for SLE-derived PBLs. Similar results were obtained by proliferation assays. These findings underscore the value of the CellScan in monitoring specific cell response to nucleosomal antigen in SLE patients, thus providing a means of monitoring disease activity and response to therapy (Gilburd et al.).

*Atherosclerosis* is a histopathologic process characterized by gradual accumulation of lipids in the vessel wall with subsequent recruitment of smooth muscle cells, T-lymphocytes and additional cellular components. In recent years, considerable data has been accumulated regarding the involvement of the immune system in atherogenesis. This hypothesis was suggested by the presence of immunocompetent cells and immunoglobulin deposition in the vicinity of the plaques, and by successful immunomodulation of the atherosclerotic process.

OxLDL, which has been considered to be an acceptable immunogen, is suspected to be one of the target autoantigens that trigger the "ongoing" local inflammatory reaction. The CellScan has been used to monitor the reactivity of PBLs to oxLDL in atherosclerotic patients and healthy controls. Exposure of PBLs to high concentration of oxLDL has lead to a decrease in FI and an increase in FP in both atherosclerosis and control groups (Zurgil et al. 1999a). Such fluorescent changes have been established to be well correlated with apoptotic processes (Zurgil et al. 2000), suggesting an apoptotic response to the cytotoxic damage of oxLDL. In low concentrations of oxLDL, however, FP decreased and FI increased in atherosclerosis-derived PBLs, suggesting cellular stimulation. This dual effect of oxLDL, namely depolarization at low dose and hyperpolarization at a higher dose, was evident in patients with active ischemic heart disease, but not in the control group (Zurgil et al. 1999a). One of the explanations suggested is that lymphocytes of atherosclerotic patients are more resistant to apoptosis by oxLDL damage, therefore allowing their continuous role in the inflammatory response. Nevertheless, the FP and FI measurements provide a simple, non-invasive tool of assessing atherosclerosis (Zurgil et al. 1999a).

Other target autoantigens suspected of generating an immune response in the atherosclerotic plaque are human β2 glycoprotein and heat shock proteins—HSP 65, and lysophophatidylcholine (LPC). *In vitro* stimulation of PBLs of patients with unstable (UA) and stable angina (SA) pectoris with LPC resulted in significant activation as manifested by the proliferation assay as well as the CellScan FP measurements. Similar results were obtained following incubation of PBLs derived from UA and SA patients with β2 glycoprotein and mycobacterial HSP65. Such findings were not observed in PBLs of the control group. These findings demonstrate once more the possibility of using the CellScan as a diagnostic and clinical-assessment tool (Gilburd et al. in press).

### Clinical pharmacology—monitoring drug efficacy and drug allergy

Adverse reactions to pharmaceutical and diagnostic products constitute a major hazard in the practice of medicine, and are responsible for substantial morbidity and cost. In the vast majority of drug reactions, diagnosis is based on clinical parameters and lacks objective confirmation. Currently, there are no highly specific tests that are predictive either of the capacity of novel compounds (drugs) to induce allergic reactions, or of the susceptibility of individuals to experience allergic reaction. Two of the conventional *in vitro* assays used to diagnose drug-induced skin reactions are the histamine-release test and the interferon-γ secretion test.

Using the CellScan, FP reduction in PBLs following incubation with an allergenic drug has been monitored. The sensitivity and specificity of the CellScan in detecting drugs responsible for skin reactions have been compared to those of the histamine-release test and the interferon-γ secretion test. The analysis of the detective ability of the three tests, which was based on clinical suspicion, has disclosed a low sensitivity (41%) and specificity (69%) for the histamine-release test, a relative high sensitivity (83%) and a moderate specificity (76%) for the Interferon-γ test, and a high sensitivity (87%) and a moderate-low specificity (53%) for the CellScan. The CellScan method has been shown to have the highest sensitivity, while the interferon-γ secretion test has had the highest specificity for the detection of the culprit drug. However, the analysis of 105 normal control sera has disclosed a high specificity of 94% for the CellScan. These preliminary findings illustrate the CellScan as a promising new method for the detection of drugs responsible for adverse skin reactions (Goldberg et al. in press).

Methotrexate (MTX) has been found to be the preferred drug for RA patients. However, not all RA patients respond to MTX, and those who do experience an overall plateau of clinical response only after six months of treatment. FP analysis has been used as a method to predict the efficiency of MTX treatment in RA patients through an *in vitro* model. Considering the RA profile of clonal-expanded CD4+T-cells, PHA-activated mononuclear cells taken from healthy donors were incubated with different concentrations of MTX. The MTX-immunosuppressive effect was tested by FP measurements of the pre-incubated and FDA-stained cells as a marker of activation or suppression. In healthy activated mononuclear cells, it has been found that MTX, through its early incubation period, interferes with the activation signal obtained by PHA and exerts an apoptotic signal, which is noted by the increase in FP. This finding was confirmed by PI assay, annexin V assay, and flow cytometry cell-cycle analysis, all demonstrating the MTX cytotoxic and proliferation-inhibiting effect.

FP analysis of RA patients-derived mononuclear cells has shown a pattern of depolarization, as a measurement of lymphocyte activation, and then hyperpolarization as a measurement of an immunosuppressive effect. These finding demonstrate the great potential of FP measurements as a tool for predicting the efficiency of MTX in RA-patients, as well as for making timing and dosage decisions (Herman et al. 2003).

A major problem in clinical oncology is the observation that neoplastic diseases classified identically, according to their histopathological characteristics, often differ widely in their sensitivity to drugs. Current cancer treatment is based on the results of random trials preformed on patients with similar types and stages of the disease. For reasons not well understood, only a limited number of patients respond to a given chemotherapy protocol. In view of this phenomenon, various chemosensitivity assays have been developed in order to select the most effective chemotherapy regimen and to avoid the toxicity of potentially ineffective drugs. Clinical trials using these assays, however, have not had a significant impact on patient survival.

The CellScan has been used to monitor FI and FP changes in fluorescently labeled cancer cells, in order to investigate the effect of anti-neoplastic drugs on human breast cancer cell-lines.

It has been shown that an increase in FP and a concomitant decrease in FI are well correlated with the cytotoxic effect of navelbine on T80 human breast cancer cell-lines. This correlation was confirmed by the number of living cells following exposure to the drug, as well as by apoptosis levels measured by the Annexin V test, attributing the hyperpolarization observed to drug-induced apoptosis.

Fluorescent hyperpolarization and reduced fluorescent intensity were not observed in drug-resistant cells, as was demonstrated in T47D human breast cancer cell-lines following exposure to 5FU. These findings further establish the ability of the CellScan to differentiate between drug sensitivity and resistance in cancer-cells.

Being independent of proliferation or colony-formation assessment, the CellScan test does not require the difficult stage of tumor culturing, common to many drug-sensitivity assays. This characteristic enables more samples to be tested, each in a shorter assay-time, thus making the CellScan a test of great potential for the prediction of response and refractoriness of neoplastic tumors to chemotherapy (Schiffenbauer et al. 2002).

### Summary

The CellScan apparatus may be used as a powerful tool for cytometric studies. Its ability to monitor fluorescence changes in individual cells, as well as kinetics of intracellular processes, creates a variety of applications in cell biology, immunology, cancer research and clinical pharmacology. The simplicity and rapidity of the CellScan make it a promising new method in both basic and clinical research.

### References

Afrimzon E, Zurgil N, Shafran Y, Sandbank J, Orda R, Lalchuk S, Deutsch M. 2004. Monitoring of intracellular enzyme kinetic characteristics of peripheral mononuclear cells in breast cancer patients. Cancer Epidemiol Biomarkers Prev 13:235–241.

Cercek L, Cercek B. 1997. Application of the phenomenon of changes in structuredness of cytoplasmic matrix (SCM) in the diagnosis of malignant disorders: A review. Eur J Cancer 13:903–915.

Cohen CJ, Denkberg G, Schiffenbauer YS, Segal D, Trubniykov E, Berke G, Reiter Y. 2003. Simultaneous monitoring of binding to and activation of tumor-specific T lymphocytes by peptide-MHC. J Immunol Methods 9346:1–14.

Deutsch M, Ron I, Weinreb A, Tirosh R, Chaitchik S. 1996. Lymphocyte fluorescence polarization measurements with the CellScan system: Application to the SCM cancer test. Cytometry 23:159–165.

Deutsch M, Zurgil N, Kauffman M, Berke G. 2000. Fluorescence polarization as an early measure of T-lymphocyte stimulation. Methods Mol Biol 134:221–242.

Deutsch M, Kaufman M, Shapiro H, Zurgil N. 2000. Analysis of enzyme kinetics in individual living cells utilizing fluorescence intensity and polarization measurements. Cytometry 39:36–44.

Eisenthal A, Marder O, Dotan D, Baron S, Lifschitz-Mercer B, Chaitchik S, Tirosh R, Weinreb A, Deutsch M. 1996. Decrease of intracellular fluorescein fluorescence polarization (IFFP) in human peripheral blood lymphocytes undergoing stimulation with phytohaemagglutinin (PHA), Concanavalin A (Con A), Pokeweed mitogen (Pwm) and anti-CD3 antibody. Biol Cell 86:145–150.

Eisenthal A, Marder O, Lifschitz-Mercer B, Skornick Y, Tirosh R, Weinreb A, Deutsch M. 1996. Inhibition of mitogen-induced changes in intracellular fluorescein fluorescence polarization of human peripheral blood lymphocytes by colchicine, vinblastine and cytochalasin B. Cell Struct Funct 21:159–166.

Eisenthal A, Marder O, Lifschitz-Mercer B, Skornick Y, Fixler D, Tirosh R, Avtalyon R, Deutsch M. 1997. Influenza A virus affects the response of human peripheral blood mononuclear cells to phytohaemagglutinin A by altering the cytoskeleton. Pathobiology 65:69–74.

Eisenthal A, Marder O, Lifschitz-Mercer B, Skornick Y, Tirosh R, Irlin Y, Avtalyon R, Deutsch M. 1997. Infection of K562 cells with Influenza A virus increases their susceptibility to natural killer lysis. Pathobiology 65:331–340.

Fixler D, Tirosh R, Eisenthal A, Marder O, Irlin Y, Lalchuk S, Deutsch M. 1997. Monitoring of effector and target cell stimulation during conjugation by fluorescence polarization. Biol Cell 89:443–452.

Fixler D, Tirosh R, Eisenthal A, Lalchuk S, Marder O, Irlin Y, Deutsch M. 1998. Prelytic stimulation of target and effector cells following conjugation as measured by intracellular fluorescein fluorescence polarization. J Biomed Optics 3:312–325.

Gilburd B, Shovman O, Zandman-Goddard G, Shiffenbauer YS, Trubniykov E, Severin M, Shoenfeld Y, Autoantigen cell activation for rapid diagnosis of different autoimmune disorders: Comparison of proliferation assays with a static cytometer (CellScan). In press.

Goldberg I, Gilburd B, Szyper Kravitz M, Kivity S, Ben Chaim B, Klein T, Brenner S, Shoenfeld Y, A novel system to diagnose cutaneous adverse drug reactions employing the CellScan—comparison with histamine releasing test and INF-γ releasing test: Preliminary Report In Press.

Herman S, Zurgil N, Langevitz P, Ehrenfeld M, Deutsch M. 2003. The Induction of apoptosis by methotrexate in activated lymphocytes as indicated by fluorescence hyperpolarization: A possible model for predicting methotrexate therapy for rheumatoid arthritis patients. Cell Struct Funct 28:113–122.

Kaplan MR, Trubniykov E, Berke G. 1997. Fluorescence depolarization as an early measure of T-lymphocyte stimulation. J Immunol Methods 201:15–24.

Klein O, Lin S, Embon O, Sazbon A, Zidan J, Kook AI. 1999. An approach for high sensitivity detection of prostate cancer by analysis of changes in structuredness of the cytoplasmic matrix of lymphocytes specifically induced by PSA-ACT. J Urol 161:1994–1996.

Klein O, Linn S, Hadari A. 2002. An approach for high sensitivity detection of breast cancer by analysis of changes in structuredness of the cytoplasmic matrix of lymphocytes specifically induced by a specific breast tumor antigen (MUC-1/SEC). Breast 11:137–143.

Klein O, Linn S, Davidson C, Hadary A, Shukha A, Zidan J, Eitan A, Kook AI. 2002. Early detection of malignant process in benign lesions of breast tumor by measurements of changes in structuredness of cytoplasmic matrix in circulating lymphocytes (SCM test) reinduced *in vitro* by specific tumor antigen. Breast 11:478–483.

Marder O, Shoval S, Eisenthal A, Fireman E, Skornick Y, Lifschitz-Mercer B, Tirosh R, Weinreb A, Deutsch M. 1996. Effect of interleukin-1$\alpha$, interleukin-1$\beta$ and tumor necrosis factor-$\alpha$ on the intracellular fluorescein fluorescence polarization of human lung fibroblasts. Pathobiology 64:123–130.

Merimsky O, Deutsch M, Tirosh R, Wohl I, Weinreb A, Chaitchik S. 1996. Detection of colon cancer by monitoring the intracellular fluorescein fluorescence polarization changes in lymphocytes. Cancer Detect Prev 20:300–307.

Merimsky O, Kaplan B, Deutsch M, Tirosh R, Weinreb A, Chaitchik S. 1997. Detection of melanoma by monitoring the intracellular fluorescein fluorescence polarization changes in lymphocytes. Cancer Detect Prev 21:167–177.

Rahmani H, Deutsch M, Ron I, Gerbat S, Tirosh R, Weinreb A, Chaitchik S, Lalchuk S. 1996. Adaptation of the CellScan technique for the SCM test in breast cancer. Eur J Cancer 32A:1758–1765.

Ron IG, Deutsch M, Tirosh R, Weinreb A, Eisenthal A, Chaitchik S. 1995. Fluorescence polarisation changes in lymphocyte cytoplasm as a diagnostic test for breast carcinoma. Eur J Cancer 31A:917–920.

Ron IG, Deutsch M, Merimsky O, Tirosh R, Rachmani H, Kaufman M, Weinreb A, Chaitchik S. 1996. Fluorescence polarization changes in the lymphocytic cytoplasm in the various stages of breast cancer. Oncol Rep 3:197–199.

Schiffenbauer Y, Trubniykov E, Zacharia B, Gerbat S, Rehavi Z, Berke G, Chaitchik S. 2002. Tumor sensitivity to anti-cancer drugs predicted by changes in fluorescence intensity and polarization *in vitro*. Anticancer Res 22:2663–2670.

Sunray M, Kaufman M, Zurgil N, Deutsch M. 1999. The trace and sub-grouping of lymphocyte activation by dynamic fluorescence intensity and polarization measurements. Biochem Biophys Res Commun 261:712–719.

Sunray M, Zurgil N, Shafran Y, Deutsch M. 2002. Determination of individual cell Michaelis-Menten constants. Cytometry 47:8–16.

Zurgil N, Deutsch M, Tirosh R, Brodie C. 1996. Indication that intracellular fluorescence polarization of T lymphocytes is cell cycle dependent. Cell Struct Funct 21:271–276.

Zurgil N, Gerbat S, Langevitzh P, Tishler M, Ehrenfeld M, Shoenfeld Y. 1997. Intracellular fluorescence polarization measurements by the CellScan system: Detection of cellular activity in autoimmune disorders. Isr J Med Sci 33:273–279.

Zurgil N, Levy M, Deutsch M, Gilburd B, George J, Haratz D, Kaufman M, Shoenfeld Y. 1999. Reactivity of peripheral blood lymphocytes (PBL) to oxLDL: A novel system to estimate atherosclerosis employing the CellScan. Clin Cardiol 22:526–532.

Zurgil N, Kaufman M, Deutsch M. 1999. Determination of cellular thiol levels in individual viable lymphocytes by means of fluorescence intensity and polarization. J Immunol Methods 229:23–34.

Zurgil N, Gerbat S, Langevits P, Tishler M, Ehrenfeld M, Kaufman M, Deutsch M, Shoenfeld Y. 1999. Detection of cellular activity in autoimmune disorders by the CellScan system. In: Shoenfeld Y, editor. The decade of autoimmunity. Elsevier Science B.V. p 295–304.

Zurgil N, Schiffer Z, Shafran Y, Kaufman M, Deutsch M. 2000. Fluorescein fluorescence hyperpolarization as an early kinetic measure of the apoptotic process. Biochem Biophys Res Commun 268:155–163.

Zurgil N, Shafran Y, Fixler D, Deutsch M. 2002. Analysis of early apoptotic events in individual cells by fluorescence intensity and polarization measurements. Biochem Biophys Res Commun 290:1573–1582.

Zurgil N, Solodeev I, Gilburd B, Shafran Y, Afrimzon E, Avtalion R, Shoenfeld Y, Deutsch M. 2004. Monitoring the apoptotic process induced by oxidized low-density lipoprotein in jurkat T-lymphoblast and u937 monocytic human cell Lines. Cell Biochem Biophys 40:97–113.

